# Dietary Rosmarinic Acid Improved the Growth, Immunity and Antioxidation of *Litopenaeus vannamei*

**DOI:** 10.3390/ani16172767

**Published:** 2026-09-03

**Authors:** Yuting Xu, Shunxiao Shi, Beiping Tan, Xiaoqin Li, Xiangjun Leng

**Affiliations:** 1 National Demonstration Center for Experimental Fisheries Science Education, Shanghai Ocean University, Shanghai 201306, China; 2 Research Centre of the Ministry of Agriculture and Rural Affairs on Environmental Ecology and Fish Nutrition, Shanghai Ocean University, Shanghai 201306, China; 3 Laboratory of Aquatic Nutrition and Feed, College of Fisheries, Guangdong Ocean University, Zhanjiang 524088, China; bptan@126.com

**Keywords:** rosmarinic acid, *Litopenaeus vannamei*, growth performance, immunity, transcriptome

## Abstract

This study investigated dietary effects of rosmarinic acid (RA) on the growth and immunity of *Litopenaeus vannamei*, and performed hepatopancreatic transcriptomic analysis to explore potential underlying molecular associations. RA was added to basal diet at 0 mg/kg (RA0), 50 mg/kg (RA50), 150 mg/kg (RA150), and 450 mg/kg (RA450) to feed *L. vannamei* (1.80 ± 0.03 g) for 8 weeks. The RA450 group exhibited higher weight gain and a lower feed conversion ratio than the control (*p* < 0.05). Serum ACP and ACH50 activities in all RA-supplemented groups (RA50, RA150 and RA450), as well as AKP and LZM activities in the RA150 and RA450 groups, were significantly higher than those in the control (*p* < 0.05). The RA150 and RA450 groups also showed higher T-AOC, R-cell count, lower MDA content in hepatopancreas, and greater intestinal villus width and muscular thickness than the control (*p* < 0.05), as well as lower mortality in *Vibrio parahaemolyticus* challenge (*p* < 0.05). Transcriptomic analysis showed that DEGs in the three RA-supplemented groups were enriched in metabolism pathways such as arachidonic acid, glycosaminoglycan degradation, fatty acid biosynthesis and peroxisome. Among them, growth-related genes (*cyp2l*, *hexb*, *hacd*) and immune/antioxidant genes (*ddo*, *xdh*, *npc2*, *galst*, *gst3*) were upregulated by dietary RA. In summary, dietary RA improved the growth, immune and antioxidant functions of *L. vannamei* and its resistance to *V. parahaemolyticus*. The improvements were associated with transcriptional changes in lipid-metabolism-related pathways and immune/antioxidant-related genes. These findings provide a theoretical basis for the application of rosmarinic acid as a green additive in the sustainable aquaculture of *L. vannamei*.

## Simple Summary

Rosmarinic acid (RA) is a polyphenolic acid widely existing in the plants of the Lamiaceae and Boraginaceae families, which exhibits a variety of biological activities, such as antioxidant and anti-inflammatory effects. This study investigated the effects of dietary RA supplementation on the growth performance, immune and antioxidant enzyme activities, hepatopancreatic and intestinal histology, and disease resistance of *Litopenaeus vannamei*. A transcriptomic analysis of hepatopancreas was also conducted to explore potential underlying molecular associations. The results revealed that dietary RA improved the growth performance, immune and antioxidant functions of *L. vannamei*, and increased resistance to *Vibrio parahaemolyticus*. Transcriptomic analysis indicated that RA may affect multiple biological processes, including the arachidonic acid metabolism, fatty acid biosynthesis, and peroxisome pathways, among others. The findings will direct the application of RA in aquafeeds to promote the green and sustainable development of the aquaculture industry.

## 1. Introduction

*Litopenaeus vannamei* has become the world’s most important farmed shrimp species due to its high economic value and strong adaptability. As the scale and density of aquaculture increase, the shrimp industry is facing various challenges such as the shortage of genetic resources, the scarcity of feed ingredients, the frequent outbreaks of disease, and the treatment of aquaculture wastewater [1,2]. In response to the above issues, the concept of green and healthy aquaculture has gradually become an industry consensus. Phytogenic additives have demonstrated positive effects in promoting growth, enhancing immunity, and improving disease resistance in *L. vannamei* culture, emerging as a key direction for antibiotic replacement and sustainable aquaculture development [3].

Rosmarinic acid (RA) is a naturally multifunctional polyphenolic acid primarily existing in the plants of Lamiaceae and Boraginaceae families. RA exhibits extensive in vitro antioxidant activity by scavenging superoxide anions and hydroxyl radicals [4], preventing the formation of lipid hydroperoxides, and inhibiting lipid peroxidation and rancidity [5]. RA can also exert potent anti-inflammatory effects by reducing the formation of arachidonic acid [6]. In addition, RA can inhibit the activity of hyaluronidase and β-hexosaminidase, maintain the integrity of tissue barriers, enhance the resistance to pathogens, and alleviate the damage caused by excessive inflammation, thereby improving the growth performance and stress tolerance [7]. In vitro studies have also shown that RA exhibited pharmacological activities such as antibacterial, antiviral, antidiabetic, antitumor, and neuroprotective effects [8]. These well-documented biological properties make RA particularly suitable for addressing the core physiological challenges in intensive *L*. *vannamei* aquaculture. The potent antioxidant activity of RA can neutralize harmful free radicals and protect hepatopancreatic cells from oxidative damage. Furthermore, the direct antibacterial activity of RA can also provide defense against bacterial infections causing significant economic losses in shrimp farming.

RA has been shown to improve growth performance and exert immunomodulatory effects in livestock and poultry [9,10]. However, relatively few studies have been conducted for aquatic animals. Dietary supplementation with 1 g/kg RA preparation (5% RA) significantly increased the survival of bullfrogs (*Lithobates catesbeiana*) and downregulated the mRNA expression of intestinal inflammation-related genes, including *tlr5*, *myd88*, *tnfα*, *il1β*, *cxcl8* and *cxcl12* [11]. The addition of 400, 600, and 800 mg/kg RA to feed significantly increased serum ACH50, lysozyme, and total immunoglobulin activity of goldfish (*Carassius auratus*) [12]. RA’s immunomodulatory effects have been proved, but its specific role in modulating the “lipid-metabolism-immunity crosstalk” in crustaceans remains unexplored.

RA has been demonstrated to exhibit potent anti-inflammatory, antioxidant, and antibacterial activities in some animals. We hypothesized that dietary RA supplementation could improve the growth performance, immune responses, and antioxidant capacity of *L. vannamei* by reprogramming hepatopancreatic lipid metabolism and activating specific immune-related signaling pathways. In this study, graded levels of RA (50, 150, and 450 mg/kg) were added to the basal diet to feed *L. vannamei* to investigate the effects on the growth performance and immune function. The hepatopancreas, a central organ for digestion, nutrient absorption, and innate immunity in shrimp, was collected for transcriptomic analysis to provide important insights into the molecular mechanisms underlying the physiological responses of shrimp to RA. The outcomes of this research will guide the application of RA in aquaculture feeds.

## 2. Materials and Methods

### 2.1. Ethical Statement

All the procedures for handling animals involved in this experiment were in accordance with the regulations of the Experimental Animal Ethics Committee and the Institutional Animal Care Committee of Shanghai Ocean University (Approval No.: SHOU-DW-2025-181).

### 2.2. Experimental Design and Diets

Based on the nutritional requirements of *L. vannamei*, a basal diet (the control, RA0) was formulated with fish meal inclusion of 180 g/kg fish meal, 41% crude protein, and 7% crude lipid. Based on the previous reports [11,12], RA preparation (Beijing Mreda Technology Co., Ltd., Beijing, China, purity ≥ 97%, CAS: 20283-92-5) was supplemented to the basal diet at levels of 0, 50, 150, and 450 mg/kg diet, respectively, to formulate four isonitrogenous and isolipidic diets, designated as RA0 (control), RA50, RA150, and RA450 groups. The measured RA levels in diets were 2.79, 39.88, 121.46, and 365.71 mg/kg, respectively. The ingredient composition and proximate composition are shown in Table 1.

### 2.3. Experimental Animals and Daily Management

Shrimp larvae were obtained from Shanghai Zhanxian Fisheries Professional Cooperative (Shanghai, China) and acclimated in concrete pools at the Binhai Aquaculture Base of Shanghai Ocean University. The initial water salinity was 10‰, and the acclimation period lasted 5 weeks. During this period, shrimp were fed flakes and powdered feed initially, followed by crumbled feed in the middle stage and commercial pellet feed in the final stage. The salinity was gradually reduced to 0.6‰. A total of 640 healthy shrimp, with an initial body weight of 1.80 ± 0.07 g, were randomly allocated to 16 cages (1.0 m × 1.2 m × 1.0 m) at a density of 40 shrimp per cage in still water with continuous aeration. The trial included four treatments, each with four repetitions.

Throughout the feeding interval, all shrimp were nourished four times daily at 07:00, 11:00, 17:00, and 23:00. The daily feeding rate was set at 3–8% of the total shrimp biomass per cage with a higher rate in the early stage and a lower one in the late stage. Considering the feeding habits of *L. vannamei*, the feed allocated to the morning and night feeding accounted for approximately 60% of the total. Feeding rates were adjusted dynamically according to the actual feeding response, weather and water temperature.

Continuous aeration was provided 24 h throughout the whole period. Waste siphoning and partial water exchanges were conducted once weekly in the initial weeks, and every 3–4 days in the late weeks. The 56-day feeding trial was conducted under strictly controlled water quality conditions: temperature 26–32 °C, salinity 0.5‰−1.0‰, dissolved oxygen ≥ 5.2 mg/L, pH 7.6–8.7, nitrite nitrogen ≤ 0.05 mg/L, and total ammonia nitrogen ≤ 0.1 mg/L.

### 2.4. Sample Collection

At the end of the 56-day feeding trial, all shrimp were fasted for 24 h. Before sampling, the shrimp were anesthetized by immersion in ice water. The total number and biomass of shrimp in each cage were measured to determine the growth performance parameters. Two shrimp were randomly collected from each cage and immediately stored at −20 °C until the analysis of proximate whole-body composition. Four shrimp were randomly collected from each cage to measure body length, body weight, hepatopancreas weight, and eviscerate muscle weight individually to calculate the body indices. For the measurement of T-AOC, MDA and SOD, hepatopancreas samples were immediately snap-frozen in liquid nitrogen after collection and stored at −80 °C until further processing. Another two shrimp per cage were randomly sampled to dissect hepatopancreas and intestinal tissues, and then immediately fixed in Bouin’s solution for histological sectioning and morphological observation. Hemolymph was withdrawn from the pericardial cavity of each of 6 shrimp, and then centrifuged at 1398× *g* for 10 min at 4 °C. The supernatant obtained was collected for serum biochemical analysis. Additionally, two individuals were randomly sampled from each cage, and their hepatopancreas tissues were aseptically harvested into pre-labeled cryovials followed by snap-freezing in liquid nitrogen. Three samples were randomly selected from each treatment group for total RNA extraction and subsequent transcriptomic analysis by Meiji Biomedical Technology Co., Ltd., Shanghai, China.

### 2.5. Test Parameters and Methods

#### 2.5.1. Growth Performance and Body Indices

The growth performance parameters measured in this study included survival rate (SR), weight gain (WG), feed intake (FI), and feed conversion ratio (FCR), which were determined by the following formulas.

SR (%) = 100 × (final number of shrimp/initial number of shrimp);

WG (%) = 100 × (final average mass-initial average mass)/initial average mass;

FI (g/shrimp) = total feed consumption/[(initial number + final number)/2];

FCR = total feed consumed/total weight gain;

The body indices determined in this study included condition factor (CF), hepatosomatic index (HSI), and meat yield (MY).

CF (g/cm^3^) = 100 × (whole body weight/body length^3^);

HSI (%) = 100 × (hepatopancreas weight/whole shrimp weight);

MY (%) = 100 × (muscle weight/shrimp weight).

#### 2.5.2. Proximate Composition of Diets and Shrimp

Proximate composition analysis of the diets and whole shrimp was performed according to the standard methods [13]. Moisture was determined by drying samples in an oven at 105 °C to constant weight; crude ash was measured by incineration in a muffle furnace at 550 °C for 6 h; crude protein content was determined using a Kjeldahl nitrogen analyzer (Kjeltec 2300, Foss, Tecator AB, Höganäs, Sweden); and crude lipid was assayed by the chloroform–methanol extraction method.

#### 2.5.3. Serum and Hepatopancreatic Biochemical Parameters

Serum biochemical parameters included total protein (TP, A045-2-2), glucose (GLU, A154-1-1), total cholesterol (T-CHO, A111-1-1), triglycerides (TG, A110-1-1), acid phosphatase (ACP, A060-2-2), alkaline phosphatase (AKP, A059-2-2), lysozyme (LZM, A050-1-1), and alternative complement activity (ACH50, YX-E23601). Hepatopancreatic biochemical parameters included superoxide dismutase (SOD, A001-3-2), total antioxidant capacity (T-AOC, A015-3-1), and malondialdehyde (MDA, A003-1-2). ACH50 was detected using a kit from Shanghai Youxuan Biotechnology Co., Ltd., Shanghai, China, and all other indices were analyzed according to the instructions of kits from Nanjing Jiancheng Bioengineering Institute, Nanjing, China. Detailed protocols were provided in the manufacturer’s instructions, which could be accessed via the corresponding product catalogue numbers on the official websites of the two suppliers.

#### 2.5.4. Intestinal and Hepatopancreatic Histology

Hepatopancreas and intestinal tissues fixed in Bouin’s fixative were dehydrated in graded ethanol solutions, then cleared, and embedded in paraffin prior to sectioning at 5 μm. Following deparaffinization with xylene, H&E staining, and mounting with neutral balsam, tissue morphology was observed and photographed using a light microscope (Nikon YS100, Tokyo, Japan). Intestinal villus height, villus width, and muscular thickness were measured using image analysis software (ImageJ 14.0), and the counts of B cells, R cells, and F cells in the hepatopancreas were also recorded. For each measured parameter and each experimental group, twelve photographs were taken. From each photograph, five villi or five hepatopancreatic tubules were randomly measured.

### 2.6. Challenge Test

After the sample collection, the remaining shrimp were fed with their original diets for 7 days prior to *Vibrio parahaemolyticus* (VP-1, Shanghai Ocean University) challenge.

The 7-day median lethal dose (LD_50_) of *V. parahaemolyticus* for *L. vannamei* was determined in a preliminary trial, where the injected shrimp exhibited dose-dependent mortality and dead individuals showed hepatopancreatic necrosis and whitening. The 4.7 × 10^8^ CFU/mL treatment group recorded a cumulative mortality of 50.00%, based on which the LD_50_ was calculated as 1.645 × 10^7^ CFU/shrimp (equivalent to 35 μL of 4.7 × 10^8^ CFU·mL^−1^ suspension) and established as the median lethal dose for the formal challenge test. Each experimental shrimp received an intramuscular injection of 35 μL *V. parahaemolyticus* suspension between the second and third abdominal segments. For the challenge test, 30 shrimp were randomly selected per treatment, with three replicates (10 shrimp per replicate). After the 7-day challenge trial, the cumulative mortality of shrimp in different groups was statistically analyzed.

Cumulative mortality (%) = (cumulative number of dead shrimp/initial number of shrimp) × 100.

### 2.7. Transcriptome Sequencing and Analysis

Transcriptomics analysis was performed by Meiji Biomedical Technology Co., Ltd. Total RNA was extracted from hepatopancreas tissues using QIAzol Lysis Reagent (Qiagen, CAS: 79306, Venlo, The Netherlands) according to the manufacturer’s instructions. The concentration and purity of the extracted RNA were measured using a NanoDrop 2000 (Thermo Fisher, Wilmington, NC, USA). RNA integrity was assessed by agarose gel electrophoresis, and the RQN value was determined using an Agilent 5300 (Agilent Technologies, Kuala Lumpur, Malaysia).

Raw reads obtained from the Illumina platform were processed through a series of quality control procedures, and high-quality valid data (clean reads) were ultimately generated. Subsequently, the clean data were aligned to the genome of *Penaeus vannamei* (GCF_042767895.1, https://www.ncbi.nlm.nih.gov/datasets/genome/GCF_042767895.1/ (accessed on 10 October 2024)). Clean reads were assembled and analyzed using StringTie, coupled with de novo assembly and corresponding optimization processes, to finally construct a unigene database. All genes obtained from transcriptome assembly were aligned against six major databases (GO, KEGG, EggNOG, NR, Swiss-Prot, and Pfam). Functional annotation was subsequently performed against public databases including Swiss-Prot and Pfam for genes with detected expression in the unigene library. Gene expression levels were quantified as TPM values using RSEM. Differentially expressed genes (DEGs) were screened with DESeq2. To further characterize the functions of DEGs, all DEGs were subjected to GO and KEGG annotations, as well as GO and KEGG functional enrichment analysis. The average error rate of sequencing bases corresponding to the quality-controlled data is generally below 0.1%. The criterion for differential significance was set as *p*-adjust < 0.05 and |log_2_FC| ≥ 1. Meanwhile, quality assessment of the post-quality-control sequencing data showed Q20 above 99% and Q30 above 96%.

### 2.8. qRT-PCR

To confirm the authenticity of the generated RNA-seq datasets, eight significantly DEGs associated with growth performance and immune function were subjected for qRT-PCR analysis. RNA quality was confirmed with A260/A280 = 1.8–2.1 and A260/A230 > 1.8. First-strand cDNA was synthesized using the Hifair^®^ III 1st Strand cDNA Synthesis SuperMix for qPCR(gDNA digester plus) (Yeasen, 11141ES60, Shanghai, China) at a temperature of 55 °C. The fluorescent quantitative reaction system consists of 20 μL: SYBR 10 μL, 0.4 μL of each forward and reverse primer, 4 μL of cDNA, and 5.2 μL of RNase-free ddH_2_O. The reaction programme is as follows: 95 °C pre-denaturation for 30 s; denaturation at 95 °C for 5 s, annealing and extension at 60 °C for 30 s, 40 repetitions. Gene names and primer sequences are listed in Table 2. Gene expression levels relative to the reference gene were calculated using the 2^−ΔΔCt^ approach, and each sample was analyzed in triplicate.

### 2.9. Statistical Analysis

Data were expressed as the mean ± standard deviation (mean ± SD). Before parametric analysis, the normality of the residuals and homogeneity of variances were assessed using the Shapiro–Wilk test and Levene’s test, respectively. When both assumptions were satisfied, one-way analysis of variance (ANOVA) was performed using SPSS 26.0, followed by Tukey’s multiple range test for pairwise comparisons. Pearson’s correlation analysis was performed using SPSS 26.0 on the log_2_FC values of the eight genes measured by qRT-PCR and RNA-Seq, and linear-regression fitting was simultaneously conducted to obtain the coefficient of determination (R^2^). A probability level of *p* < 0.05 was considered statistically significant.

## 3. Results

### 3.1. Growth Performance

As shown in Table 3, the WG in the RA450 group was increased by 5% and the FCR was decreased by 0.07 compared with the control (*p* < 0.05). In addition, there were no significant differences in HSI, CF or MY among all the groups (*p* > 0.05).

### 3.2. Proximate Composition of Whole Shrimp

No statistically significant variations were observed in moisture, crude lipid, crude protein, and crude ash across all experimental groups (Table 4) (*p* > 0.05).

### 3.3. Serum and Hepatopancreatic Biochemical Indices

Serum ACP and ACH50 activities in the RA50, RA150 and RA450 groups, and LZM and AKP activities in the RA150 and RA450 groups were significantly higher than those in the RA0 group (Table 5) (*p* < 0.05). However, no significant differences in TP, GLU, T-CHO or TG levels were observed among all the groups (*p* > 0.05).

Hepatopancreatic T-AOC was significantly enhanced and MDA was significantly reduced in the RA150 and RA450 groups relative to the control (*p* < 0.05). Meanwhile, SOD in the RA450 group was also significantly higher than that in the control (*p* < 0.05).

### 3.4. Intestinal and Hepatopancreatic Histology

**Figure 1:**
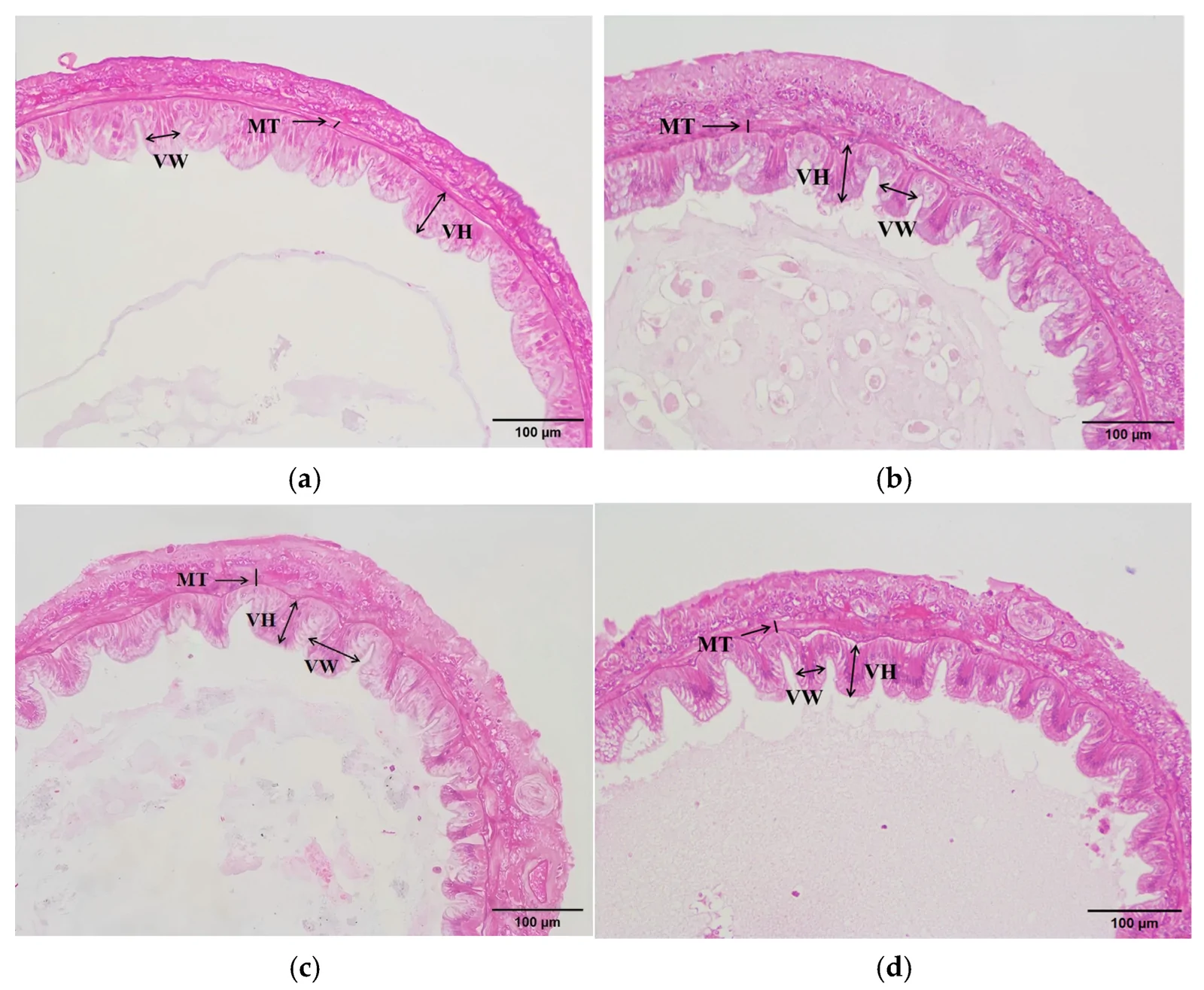
Effect of rosmarinic acid on intestinal structure of *L. vannamei* (H&E staining, 200×). Note: (**a**–**d**) represent intestinal cross-sectional micrograph of RA0, RA50, RA150 and RA450. VH: villus height; VW: villus width; MT: muscular thickness.

Intestinal villus in all groups was arranged neatly and compactly (Figure 1). Quantitative analysis of intestinal morphology demonstrated that the RA450 group had significantly higher VH than the RA0 control, and both the RA150 and RA450 groups had significantly elevated VW and MT (*p* < 0.05) (Table 6).

**Figure 2:**
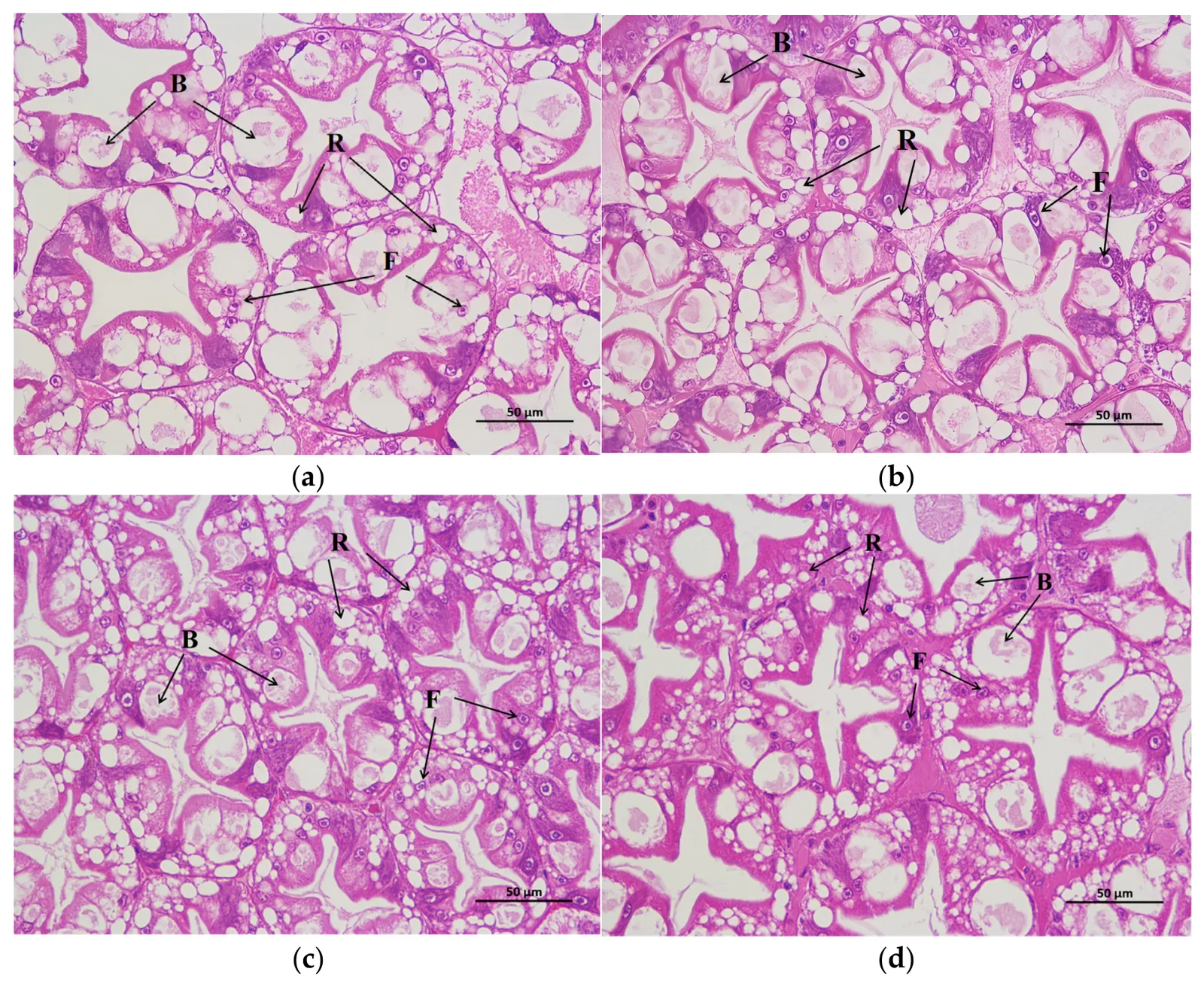
Effect of rosmarinic acid on the histological structure of hepatopancreatic tissue in *L. vannamei* (H&E staining, 400×). Note: Figures (**a**–**d**) represent the RA0 group, RA50 group, RA150 group, and RA450 group. B: Blasenzellen cells (B-cell), R: Restzellen cells (R-cell), F: fibrous cells (F-cell).

In Figure 2, the hepatopancreatic structure of *L. vannamei* was intact, clear, and neatly arranged in all groups. Significant increases in hepatopancreatic R-cell counts were detected in the RA150 and RA450 groups relative to RA0, and F-cell counts were significantly elevated in the RA450 group (*p* < 0.05) (Table 7).

### 3.5. Challenge Test

**Figure 3:**
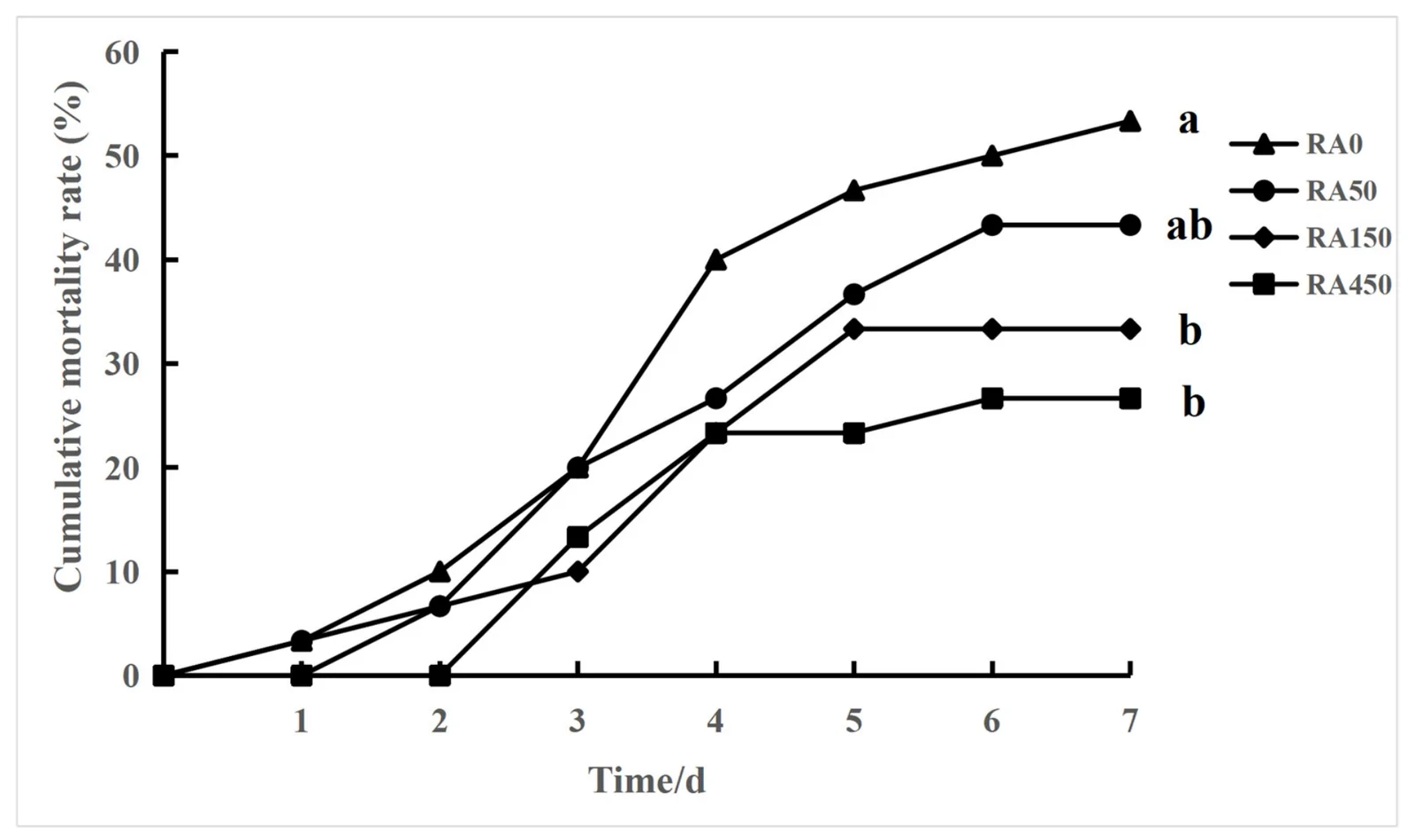
Effect of rosmarinic acid on cumulative mortality rate of *L. vannamei* following *V. parahaemolyticus* infection. Note: Different letters indicate significant differences (*p* < 0.05).

During the 7-day period after challenge with *V. parahaemolyticus*, the cumulative mortality rate of the RA0, RA50, RA150, and RA450 groups was 53.33% ± 5.77%, 43.33% ± 5.77%, 33.33% ± 15.27%, and 26.67% ± 5.77% (Figure 3). After challenge with pathogenic bacteria, *L. vannamei* fed diets containing 150 mg/kg and 450 mg/kg RA showed significantly lower mortality (*p* < 0.05).

### 3.6. Hepatopancreatic Transcriptome

#### 3.6.1. Analysis of DEGs

**Figure 4:**
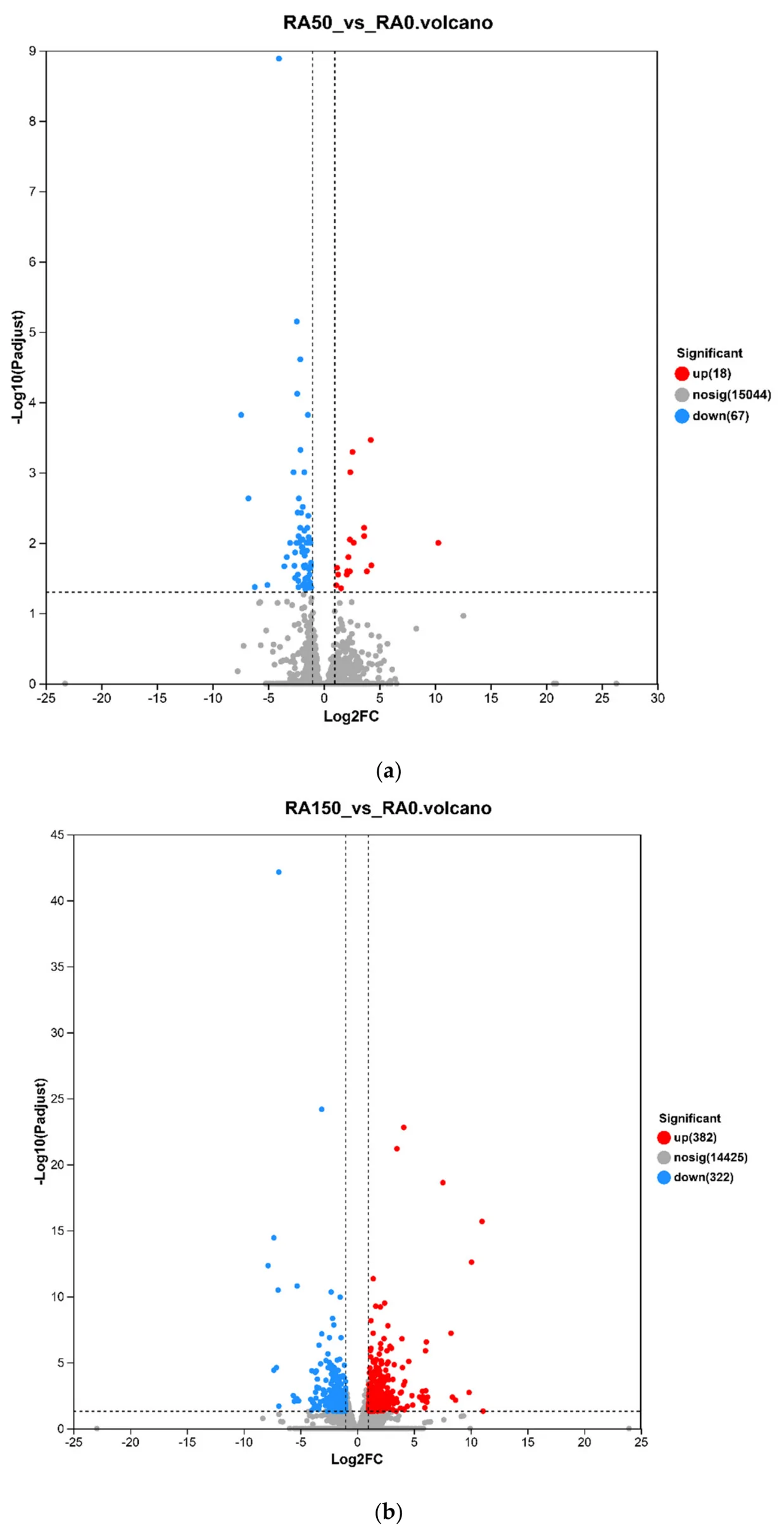

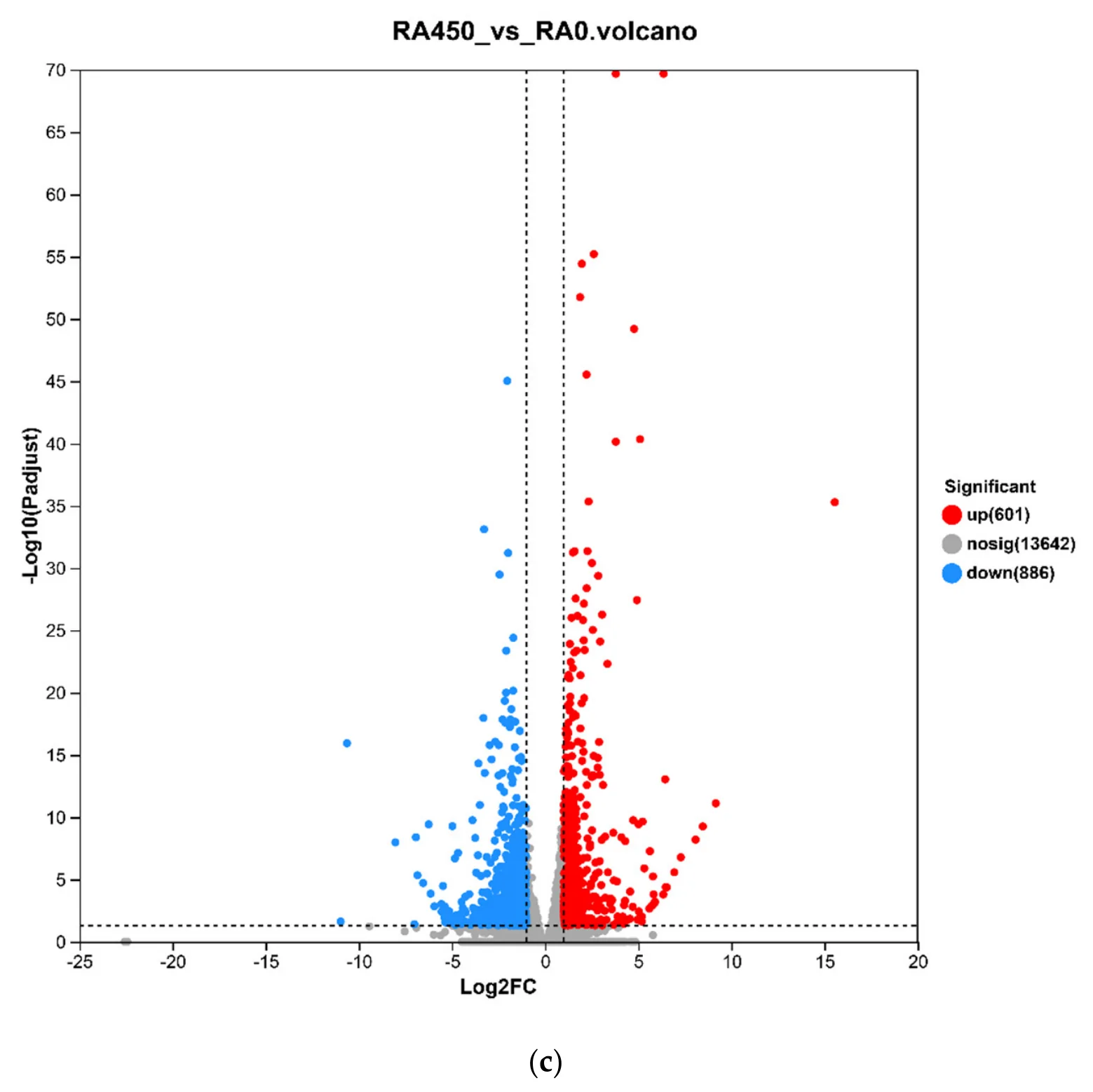
Volcano map of differentially expressed genes between rosmarinic acid groups and control groups. (**a**) RA50 vs. RA0; (**b**) RA150 vs. RA0; (**c**) RA450 vs. RA0.

As shown in Figure 4, compared with the control group, 85 DEGs were identified in the RA50 group (18 upregulated and 67 downregulated), 704 DEGs in the RA150 group (382 upregulated and 322 downregulated), and 1487 DEGs in the RA450 group (601 upregulated and 886 downregulated).

#### 3.6.2. GO Functional Annotation and Enrichment Analysis of DEGs

##### GO Functional Annotations Analysis

**Figure 5:**
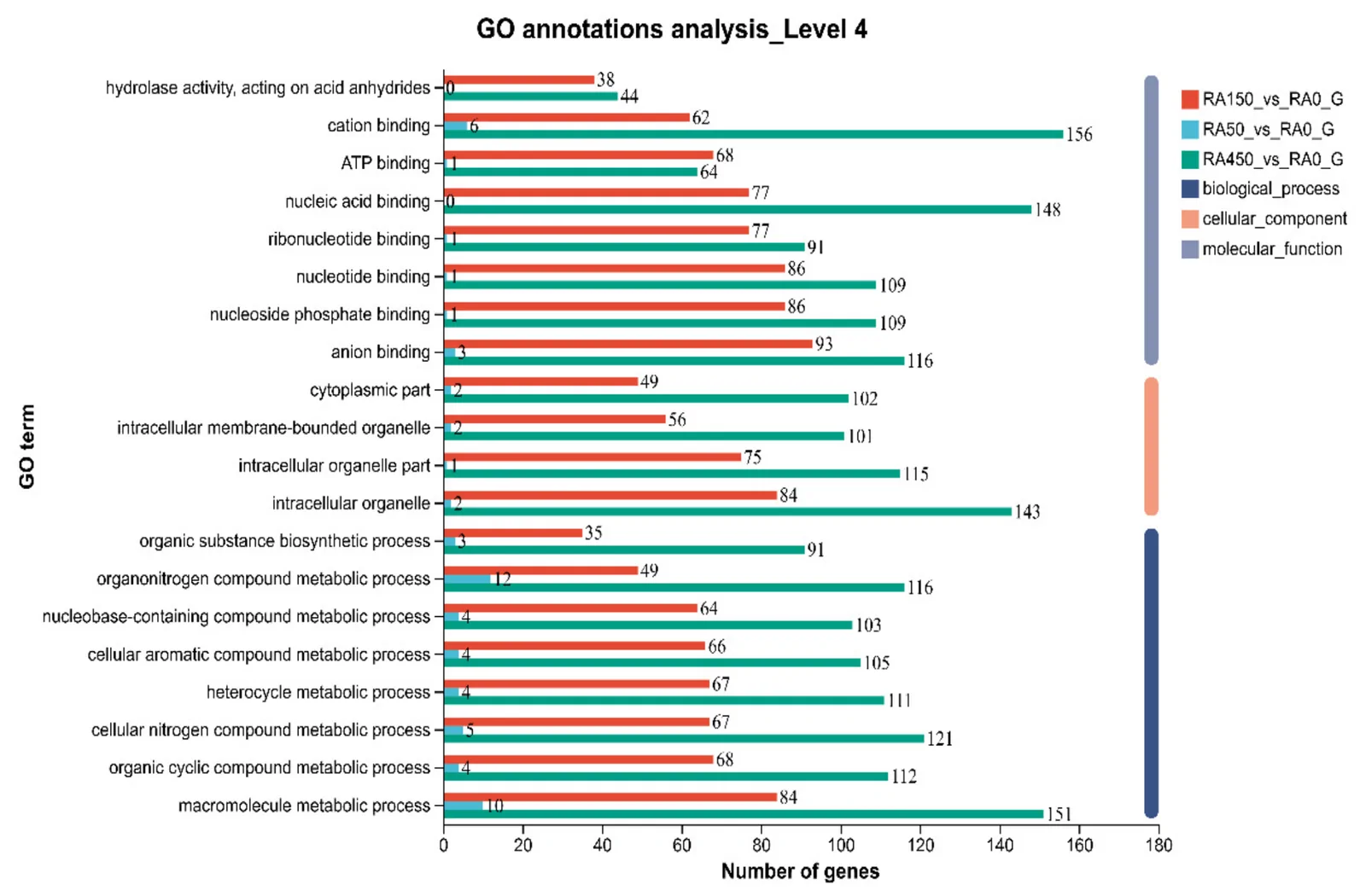
GO classification statistical histogram of hepatopancreas DEGs between rosmarinic acid groups.

According to the GO annotation analysis (Figure 5), the DEGs identified in this study were assigned to 408 second-level GO terms within three main categories: molecular function (164), cellular component (95), and biological process (149).

Within the molecular function category, the largest number of DEGs was assigned to cation binding and nucleic acid binding, followed by anion binding, nucleoside phosphate binding and nucleotide binding. In the cellular component category, the most DEGs were mapped to intracellular organelle, followed by intracellular organelle part, intracellular membrane-bounded organelle and cytoplasmic part. For the biological process category, the greatest number of DEGs was assigned to the macromolecule metabolic process, followed by the cellular nitrogen compound metabolic process, the organonitrogen compound metabolic process and the organic cyclic compound metabolic process.

##### GO Functional Enrichment Analysis

**Figure 6:**
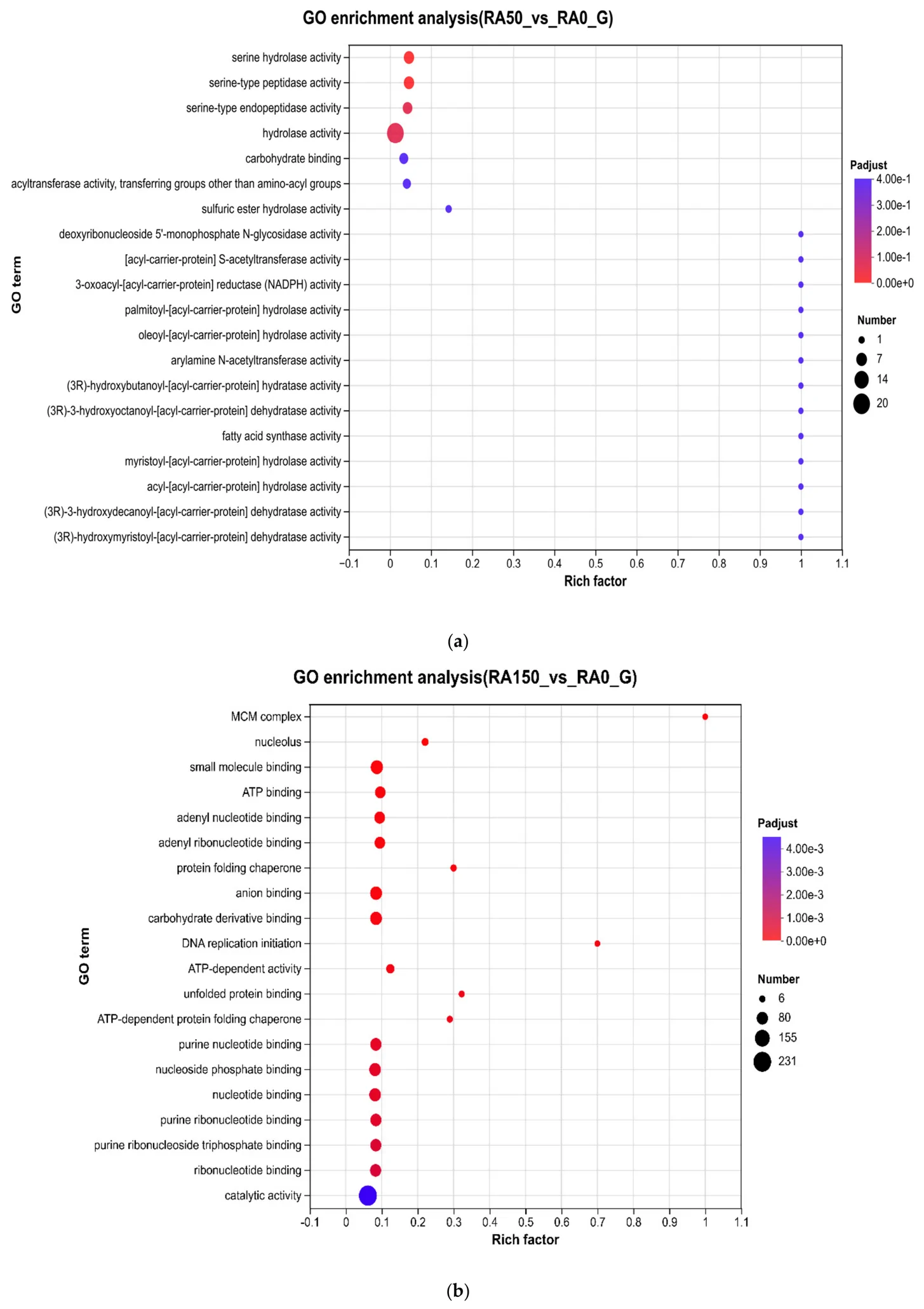

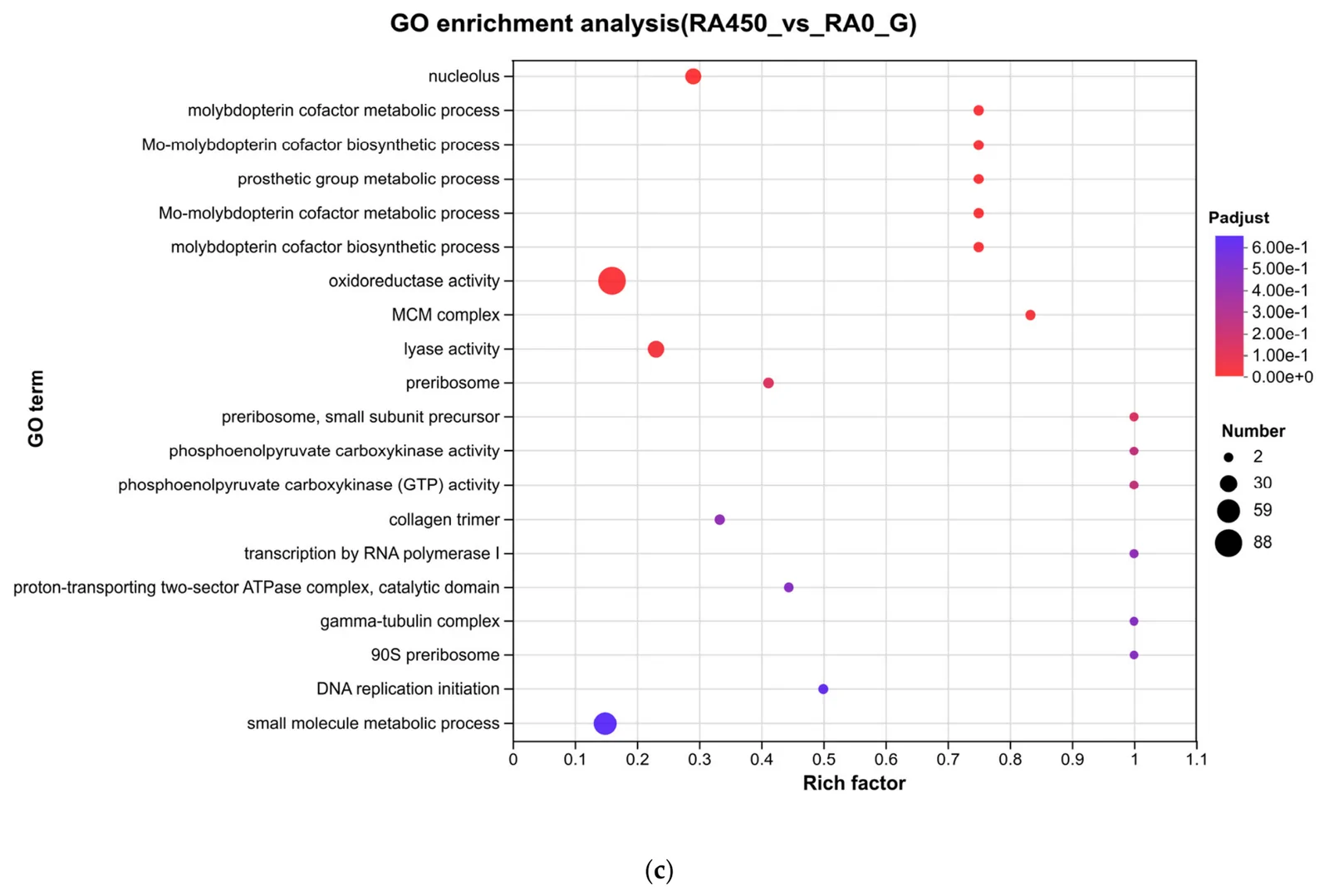
GO enrichment analysis bubble diagram of hepatopancreas DEGs between rosmarinic acid groups. (**a**) RA50 vs. RA0; (**b**) RA150 vs. RA0; (**c**) RA450 vs. RA0.

GO functional enrichment analysis was performed, as shown in Figure 6. Compared with the control group, DEGs in the RA50 group were enriched in 92 GO terms, among which two terms were significantly enriched: serine hydrolase activity and serine-type peptidase activity. DEGs in the RA150 group were enriched in 176 GO terms, among which 27 were significantly enriched. In the plot, the number of enriched DEGs ranked from high to low was as follows: catalytic activity, small-molecule binding, anion binding, carbohydrate derivative binding, nucleoside phosphate binding, and nucleotide binding. For the RA450 group, DEGs were enriched in 109 GO terms, and 9 terms were significantly enriched. The number of enriched DEGs ranked from high to low was oxidoreductase activity, lyase activity, nucleolus, the molybdopterin cofactor metabolic process, and the prosthetic group metabolic process. All three RA-supplemented groups were enriched in catalytic activity.

Notably, in contrast to the RA50 group, the RA150 and RA450 groups were both significantly enriched in nucleolus, oxidoreductase activity and MCM complex.

#### 3.6.3. KEGG Functional Annotation and Enrichment Analysis of DEGs

##### KEGG Functional Annotations Analysis

**Figure 7:**
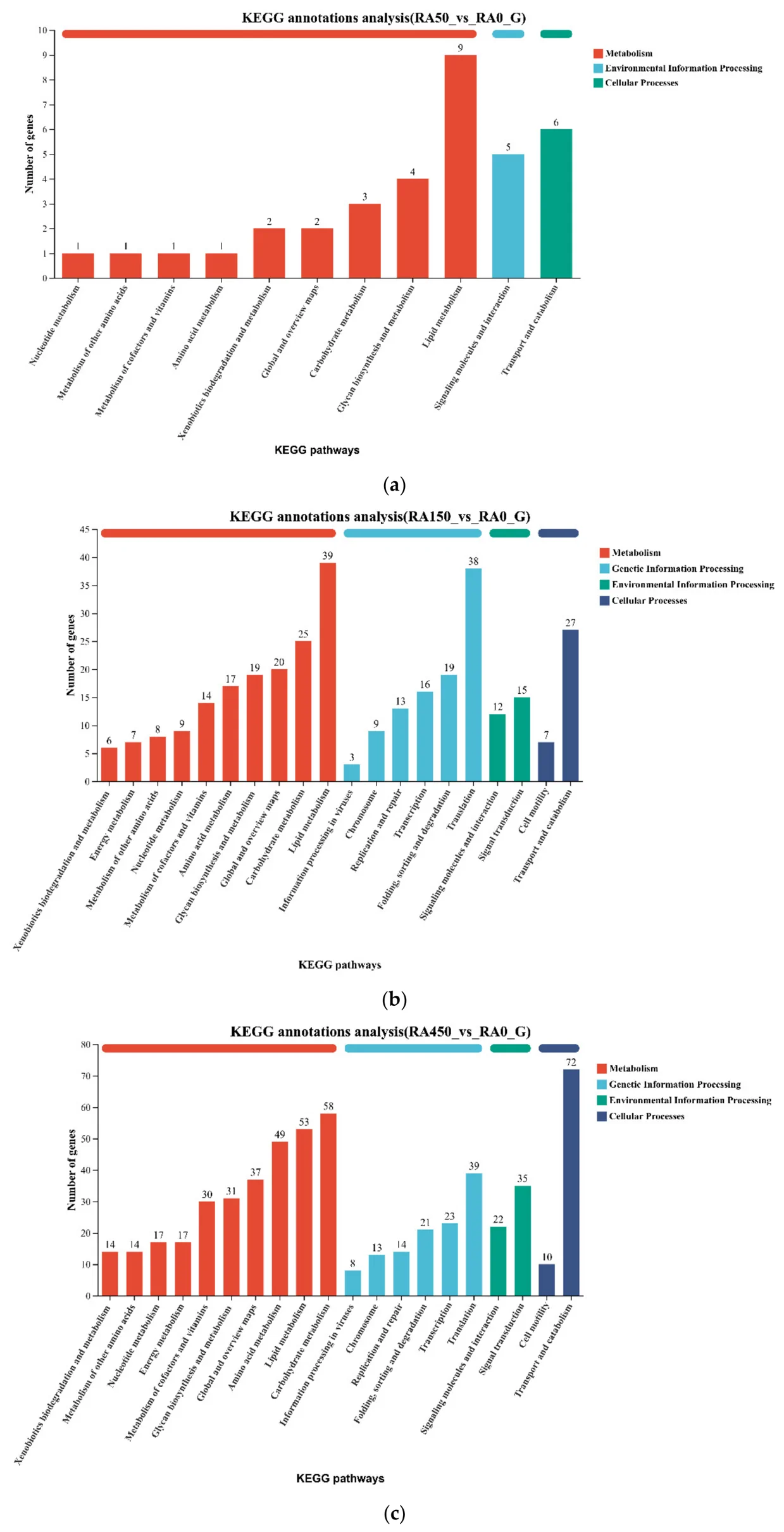
KEGG pathway classification statistical histogram of hepatopancreas DEGs between rosmarinic acid groups. (**a**) RA50 vs. RA0; (**b**) RA150 vs. RA0; (**c**) RA450 vs. RA0.

KEGG pathway annotations were performed on the DEGs identified from pairwise comparisons among the RA-supplemented groups (Figure 7).

Compared with the control group, the DEGs in the RA50 group were assigned to three major functional categories, among which the metabolism category contained the largest number of genes related to lipid metabolism, followed by transport and catabolism in the cellular process category. Additionally, in the environmental-information-processing category, DEGs were annotated solely to the signaling molecules and interaction. Both the RA150 and RA450 groups were assigned to one additional category compared with the RA50 group besides the above three categories, genetic information processing. For the RA150 group, the largest number of DEGs were also assigned to lipid metabolism in the metabolism category, followed by the translation process in the genetic-information-processing category, then transport and catabolism in the cellular process category, and finally carbohydrate metabolism in the metabolism category. For the RA450 group, the largest number of DEGs were assigned to transport and catabolism in the cellular process category. Furthermore, these DEGs were also frequently annotated to the metabolic category, mainly involving carbohydrate metabolism, lipid metabolism, and amino acid metabolism. In addition, within the genetic-information-processing category, the translation process was another frequently annotated functional type. For the DEGs in the RA150 and RA450 groups, in addition to the signaling molecules and interaction observed in the RA50 group, signal transduction was also annotated in the environmental-information-processing category.

##### KEGG Functional Enrichment Analysis

**Figure 8:**
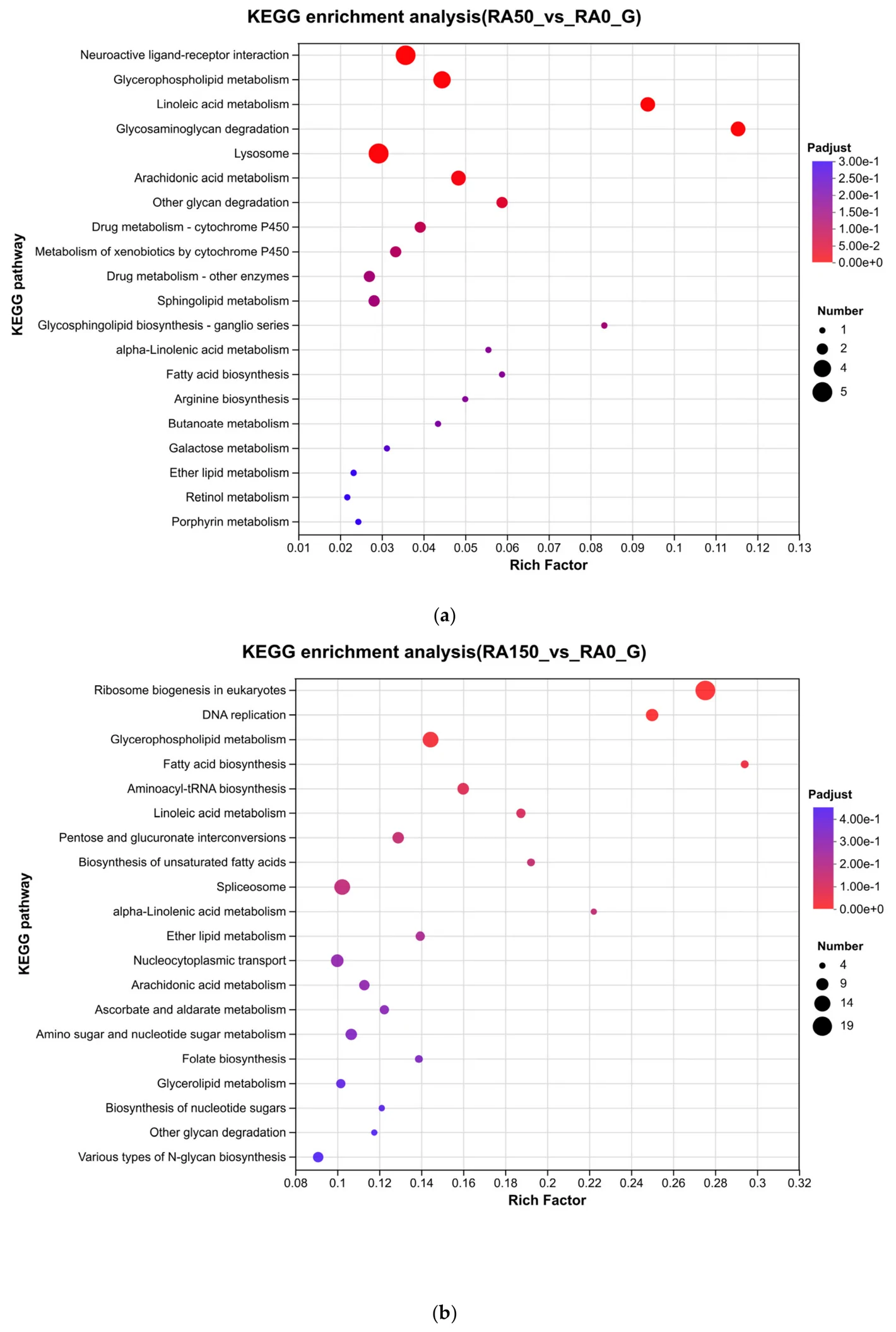

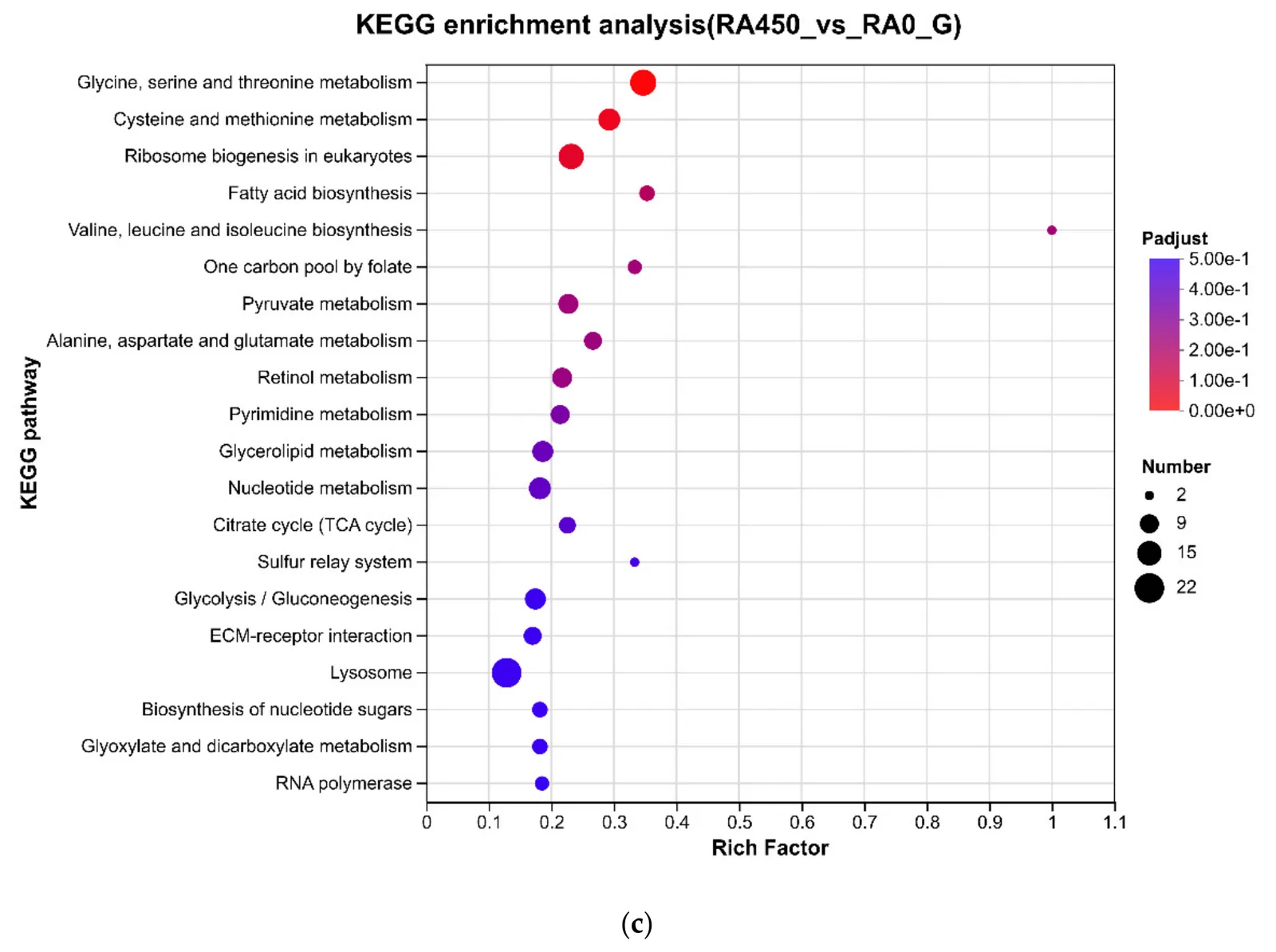
KEGG enrichment analysis bubble diagram of hepatopancreas DEGs between rosmarinic acid groups. (**a**) RA50 vs. RA0; (**b**) RA150 vs. RA0; (**c**) RA450 vs. RA0.

The KEGG enrichment results were presented as a bubble chart showing the top 20 pathways by enrichment level (Figure 8). Compared with the control group, DEGs in the RA50, RA150 and RA450 groups were enriched in 28, 111 and 120 signaling pathways, respectively, among which 7, 4 and 2 pathways were significantly enriched.

Compared with the control group, the RA50 group exhibited significant enrichment in neuroactive ligand–receptor interaction, glycerophospholipid metabolism, linoleic acid metabolism, glycosaminoglycan degradation, lysosome, arachidonic acid metabolism and other glycan degradation pathways. The RA150 group was significantly enriched in ribosome biogenesis in eukaryotes, DNA replication, glycerophospholipid metabolism and fatty acid biosynthesis pathways. The RA450 group showed significant enrichment in glycine, serine and threonine metabolism, as well as in the cysteine and methionine metabolism pathways.

Notably, the three RA-supplemented groups were all enriched in 27 common signaling pathways, including fatty acid biosynthesis, retinol metabolism, lysosome, peroxisome, drug metabolism-cytochrome P450, ether lipid metabolism, and arachidonic acid metabolism.

#### 3.6.4. qRT-PCR

**Figure 9:**
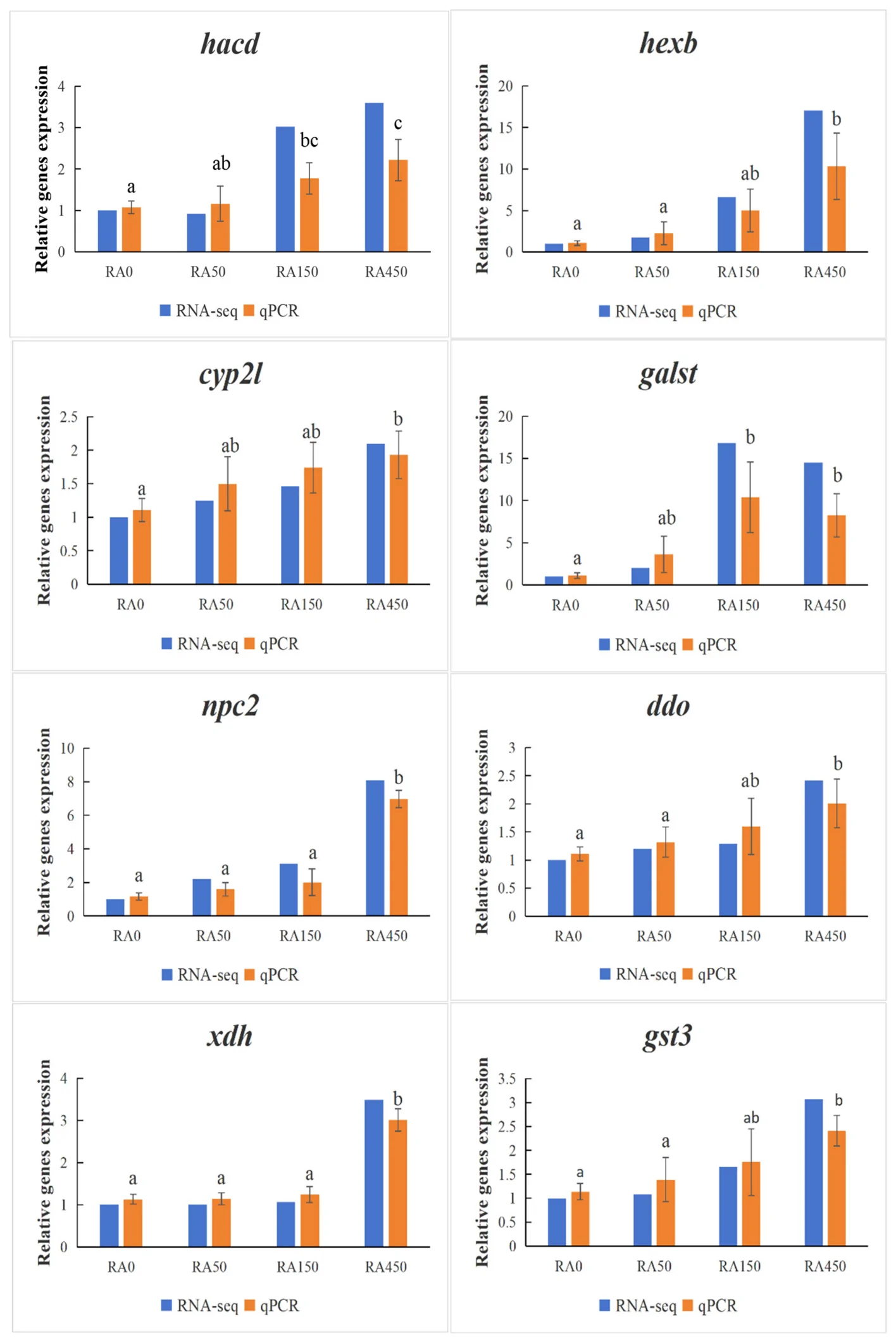
Comparison between RNA-seq and real-time fluorescence quantitative PCR. Note: Different letters indicate significant differences (*p* < 0.05).

**Figure 10:**
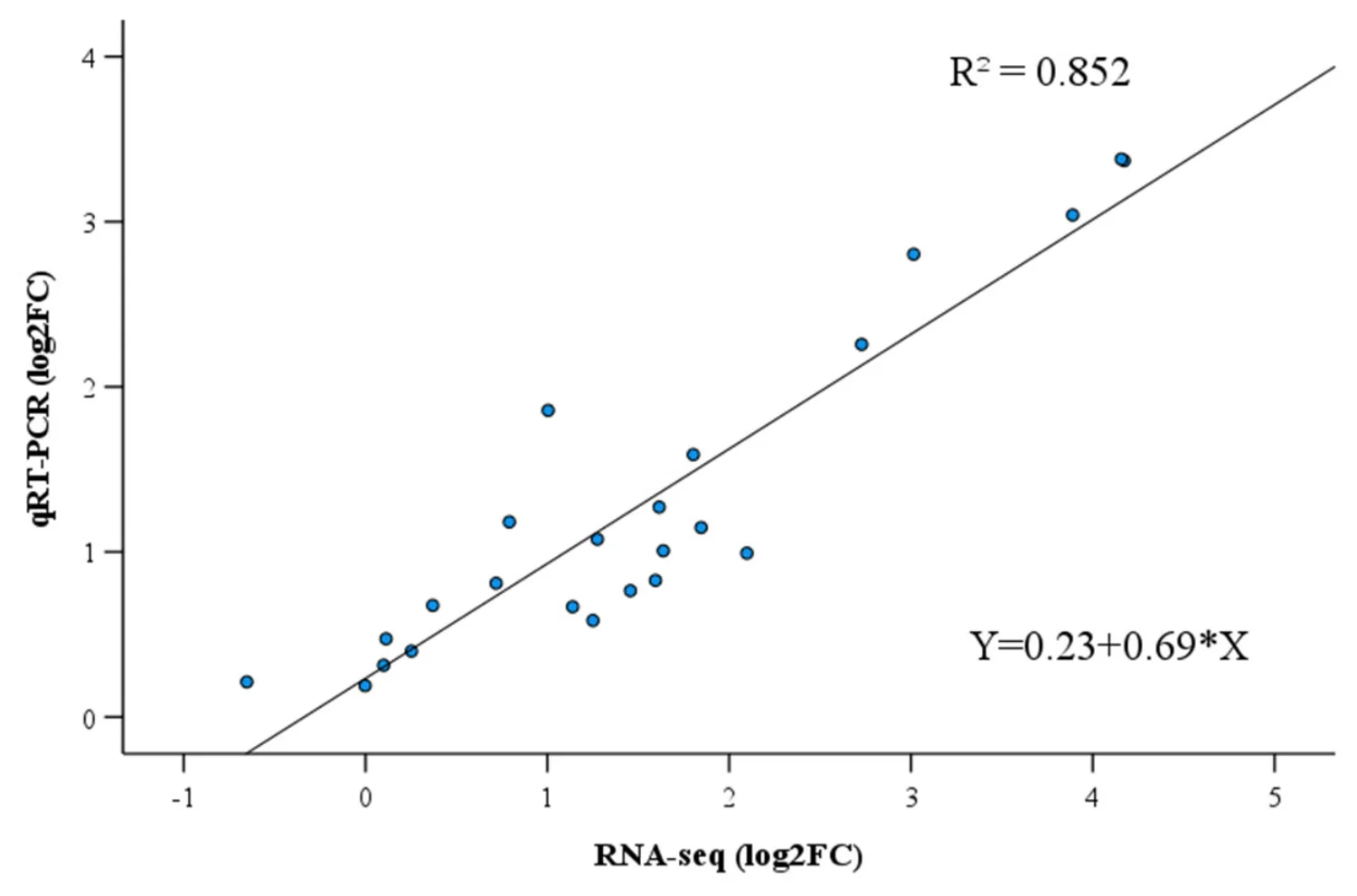
Correlation analysis of expression change values (log_2_FC) between qRT-PCR and RNA-Seq. Note: Correlation analysis was performed using the expression change values (log_2_FC) of eight selected genes between qRT-PCR and RNA-Seq. The results showed a significant positive correlation between the two methods (linear fitting method (R^2^), based on Pearson’s correlation, *p* < 0.05).

The qRT-PCR results are presented in Figure 9. Significant upregulation of *hexb*, *cyp2l*, *npc2*, *ddo*, *xdh* and *gst3* was observed in the RA450 group relative to the control (*p* < 0.05), whereas *hacd* and *galst* were significantly increased in both the RA150 and RA450 groups (*p* < 0.05). The expression patterns of these genes were consistent with the transcriptome sequencing data, verifying the reliability of the transcriptomic analysis (Figure 10).

## 4. Discussion

### 4.1. Growth Performance

RA is a polyphenolic compound. Polyphenolic compounds can improve the animal gut microbiota through their antioxidant and immunomodulatory activities, thereby promoting growth performance [14]. Dietary supplementation with 1 g/kg and 3 g/kg RA (purity ≥ 99%) significantly increased the WG and decreased the FCR in rainbow trout (*Oncorhynchus mykiss*) [15]. In goldfish, dietary supplementation with 400, 600 and 800 mg/kg RA significantly increased the WG and SGR [12]. The similar growth-promoting effects by dietary RA were also reported in common carp (*Cyprinus carpio*) [16].

In the present study, dietary supplementation with 450 mg/kg RA significantly increased the WG and reduced the FCR in *L. vannamei.* The varying inclusion level of RA may be related to the animal species with different sensitivity to RA. The growth-promoting effects of RA on aquatic animals are mainly attributed to its ability to improve intestinal health [17], as well as the strong antioxidant activity and immune defense function [18,19]. Compared with fish, shrimp possess distinct digestive and hepatopancreatic metabolic systems that may influence the absorption, transformation, and biological activity of dietary phytochemicals. Moreover, differences in dietary lipid sources, stocking density, and environmental stress conditions may contribute to the variation in physiological responses to RA supplementation. Therefore, the effective dose of 450 mg/kg identified in this study represents a species-specific response under the current experimental conditions rather than a universal optimal supplementation level.

### 4.2. Serum and Hepatopancreatic Biochemical Indices

In aquatic animals, ACP and AKP are frequently used as indicators to evaluate non-specific immunity and phagocytic activity [20]. Serum lysozyme activity is also an effective tool for assessing the baseline immune status of shrimp. ACH50 is another key indicator of non-specific immunity in shrimp. The addition of 600 mg/kg RA to the diet significantly increased serum lysozyme activity in common carp [16]. Dietary supplementation with 600 and 800 mg/kg RA significantly increased ACH50 and total Ig activity in goldfish [12]. In the present study, serum ACP, AKP, LZM activity and ACH50 in the RA150 and RA450 groups were significantly higher than those in the control group, indicating that RA enhanced the nonspecific immunity of *L. vannamei*. Studies in vertebrates indicated that RA might modulate immune responses by regulating cytokine production and inflammatory reactions; however, the underlying mechanism of RA enhancing shrimp immunity remained unclear, which needs further investigation [21,22].

MDA content is a commonly used indicator reflecting lipid peroxidation. T-AOC reflects the overall synergistic antioxidant capacity of enzymatic systems, small molecules and proteins in body [23]. Dietary supplementation with 4 g/kg RA (5% purity) significantly reduced the MDA levels of bullfrogs, suggesting that RA might alleviate high-soybean-meal-induced intestinal inflammation and oxidative stress [11]. In the present study, dietary supplementation with 150 and 450 mg/kg RA significantly reduced MDA levels and increased T-AOC in the hepatopancreas of *L. vannamei*. Additionally, the SOD activity in the 450 mg/kg RA group was significantly higher than that in the control group, indicating that RA enhanced the antioxidant capacity of *L. vannamei*.

### 4.3. Intestinal and Hepatopancreatic Histology

The mucosal lining and intestinal villus of shrimp form the first line of defense against pathogen invasion, regulating nutrient absorption efficiency and maintaining overall health [24,25,26]. Intestinal villus and the muscular layer are critical structures for nutrient digestion, absorption and intestinal barrier defense in shrimp [27]. The supplementation of 100 mg/kg RA in diets significantly increased VH in the ileum and cecum, improved the VH-to-crypt depth ratio, and enhanced the surface area and efficiency of nutrient absorption for broiler [28]. In the present experiment, dietary supplementation with 450 mg/kg RA significantly increased the intestinal VH, and the supplementation of 150 mg/kg and 450 mg/kg RA significantly increased the intestinal VW and MT of shrimp, indicating that RA may promote the growth of shrimp by enhancing the ability to digest and absorb nutrients.

The hepatopancreas is the most important center for digestion, metabolism, and immunity in shrimp; it is also a key organ for innate immunity, expressing various antimicrobial peptides, complement components, and antioxidant enzymes, and plays a crucial role in the antioxidant and immune functions of shrimp [29,30,31]. R cells in hepatopancreas are responsible for nutrient absorption and metabolism, energy storage, and detoxification. Digestive enzymes are primarily synthesized in F cells, which also produce hemolymph proteins responsible for oxygen delivery and immune responses [32,33]. B cells are responsible for the intracellular digestion of absorbed nutrients and participate in lipid emulsification and recycling [32,33]. In the present experiment, the various cellular structures were clearly distinguishable and densely arranged. The number of R cells in the RA150 and RA450 groups and the number of F cells in the RA450 group were significantly higher than those in the control group. These results indicate that RA can enhance energy storage in hepatopancreas by increasing the number of R cells [34], and the increase in F-cell number can promote the synthesis of digestive enzymes, enabling the shrimp to obtain more energy from food [35], thereby improving growth performance.

Notably, in the present study, the growth-promoting effect of RA was significant only at the highest dose (450 mg/kg), whereas the immune and antioxidant enhancements were significant at 150 mg/kg and did not further increase at 450 mg/kg. This divergent dose–response pattern suggests a potential plateau effect for immune and antioxidant parameters, where 150 mg/kg RA may have saturated the antioxidant defense system and immune activation pathways. In contrast, the growth-promoting effect appears to require a higher threshold, possibly because anabolic metabolism and nutrient utilization demand more substantial metabolic reprogramming. At 450 mg/kg inclusion, RA may prioritize energy allocation toward anabolic processes—enhanced fatty acid biosynthesis, increased R-cell proliferation for nutrient storage, and improved intestinal absorption—while the immune system reaches its maximal responsive capacity at 150 mg/kg.

### 4.4. Challenge Test

*Vibrio parahaemolyticus* is one of the most severe threats to the global shrimp aquaculture industry. Polyphenols exert direct inhibitory effects against *V. parahaemolyticus* in vitro [36]. As a natural polyphenolic acid, RA exerted inhibitory effects against a variety of bacteria, including *Enterobacteriaceae* spp. and *Pseudomonas* spp. [19]. Common carp fed a diet supplemented with 600 mg/kg RA exhibited significantly lower cumulative mortality following challenge with *Aeromonas hydrophila* compared with the control group [16]. In the present experiment, dietary supplementation with 150 and 450 mg/kg RA significantly decreased the mortality of *L. vannamei* challenged with *V. parahaemolyticus*, indicating that RA could enhance resistance against the pathogenic bacterium.

### 4.5. Transcriptome

#### 4.5.1. Arachidonic Acid Metabolism

Arachidonic acid (AA) is a critical polyunsaturated fatty acid in animal organisms, which can form various bioactive molecules via multiple enzymatic pathways, including prostaglandins and leukotrienes. These metabolites are not only involved in regulating cell differentiation, tissue development and organ function, but also exert crucial roles in inflammatory responses, cardiovascular regulation and immune modulation [37]. The CYP2J gene family, a member of the cytochrome P450 (CYP450) superfamily, catalyzes the epoxidation of AA to generate epoxyeicosatrienoic acids, representing a core component of the AA epoxidation pathway [38,39]. A high-quality chromosome-level genome of *Oryziascurvinotus* revealed a significant expansion of cytochrome P450 (CYP) detoxification gene family, which not only enhanced the detoxification capacity of this species against exogenous substances but also participated in the regulation of key physiological processes, including growth and sexual development [40]. In the present study, the *cyp2l* gene of *L. vannamei* was identified as a homolog of the CYP2J subfamily. The enhanced non-specific immune parameters (ACP, AKP, LZM, and ACH50) and increased resistance against *V. parahaemolyticus* infection observed in RA-supplemented shrimp were consistent with the potential involvement of arachidonic acid in immune regulation. Notably, high inclusion of RA (450 mg/kg) may regulate arachidonic acid metabolism through transcriptional modulation of *cyp2l*. Given that arachidonic acid-derived eicosanoids serve as important lipid mediators in crustacean inflammatory regulation and immune responses, the alteration in this pathway likely contributes to the improved immune homeostasis and disease resistance in RA-supplemented shrimp.

#### 4.5.2. Glycosaminoglycan Degradation

Glycosaminoglycans (GAGs) are widely distributed in the extracellular matrix and on cell surfaces, participating in cell-signal transduction, tissue structural support, and physiological regulation. GAG metabolism primarily relies on the synergistic action of multiple lysosomal hydrolases, which degrade GAGs into recyclable small molecules. Among these enzymes, β-hexosaminidase is one of the critical enzymes [41]. β-hexosaminidase is composed of two subunits, α and β, encoded by the *hexa* and *hexb* genes, respectively. Studies demonstrated that *hexb* gene deletion resulted in complete loss of β-hexosaminidase activity in lysosomes, and led to Sandhoff disease, a disorder characterized by neurological damage and abnormal accumulation of GAGs [41]. In the present study, in the GAG degradation pathway, the expression levels of *hexb* were upregulated in all RA-supplemented groups, with a statistically significant upregulation in the RA450 group. These results indicated that 450 mg/kg RA supplementation could mediate efficient GAG degradation by upregulating *hexb* expression, maintain the dynamic balance of GAGs, prevent histocytological structural damage caused by excessive GAG accumulation, ensure the normal execution of core physiological functions including hepatopancreatic lipid metabolism and intestinal nutrient absorption, and thus promote the growth of shrimp.

#### 4.5.3. Fatty Acid Biosynthesis

Long-chain polyunsaturated fatty acids (LC-PUFAs), particularly docosahexaenoic acid (DHA), are essential components of cell membranes, maintaining cellular function and metabolic homeostasis in fish. Dietary DHA deficiency significantly reduced the growth of *Dicentrarchuslabrax* and upregulated the expression of genes involved in DHA biosynthesis, indicating that the synthetic pathway activation incurred additional energy expenditure and consequently compromised growth [42]. (3R)-3-Hydroxyacyl-CoA dehydrogenase plays a critical role in fatty acid β-oxidation and biosynthesis, and is an essential component of the DHA biosynthetic pathway. This enzyme was encoded by the *Ehhadh* gene. *Ehhadh* knockout in zebrafish significantly inhibited DHA biosynthesis and downregulated the expression of key regulators including *srebf1* (SREBP pathway), *pparα* (PPAR pathway), and PUFA biosynthetic genes such as *fads2* and *hsd17b4*. Conversely, the overexpression of *Ehhadh* increased hepatic DHA content, and promoted PUFA biosynthesis and lipid metabolism, thereby supporting fish growth and health [43]. In the present study, the *hacd* gene in *L. vannamei* also encoded an enzyme with similar functions. Compared with the control group, the *hacd* gene expression was significantly upregulated in the RA150 and RA450 groups, indicating that dietary RA supplementation was associated with transcriptional regulation of genes involved in fatty acid metabolism in *L. vannamei*. The regulation of lipid metabolic pathways may contribute to improved physiological performance by supporting both energy allocation for growth and immune cell function. This interpretation is consistent with the improved growth performance, enhanced non-specific immune parameters (ACP, AKP, LZM and ACH50), and increased resistance against *V. parahaemolyticus* observed in RA-supplemented shrimp. However, further investigations are required to clarify whether these transcriptional changes directly affect fatty acid synthesis and lipid metabolism.

#### 4.5.4. Peroxisome

Peroxisomes regulate the development and activation of innate and adaptive immune cells via modulating fatty acid β-oxidation and ether lipid synthesis, thus fine-tuning inflammatory responses [44,45]. D-aspartate oxidase (DDO) is primarily localized to peroxisomes and regulates intracellular levels of D-amino acids, including D-aspartate and D-glutamate. DDO exerts vital functions in neurotransmission, development, hormone secretion and reproductive regulation [46,47]. Xanthine dehydrogenase (XDH) plays a crucial role in peroxisomes, primarily participating in purine metabolism and ROS generation. In *Drosophilamelanogaster*, the loss of function mutations (rosy-506) eliminated XO activity in peroxisomes and compromised the activity of intraperoxisomal catalytic enzymes, demonstrating that the *xdh* gene was indispensable for peroxisomal functional homeostasis [48]. In the present study, the expression levels of *ddo* and *xdh* were significantly increased in the RA450 group compared with the control group, indicating that high-dose RA supplementation was associated with transcriptional regulation of peroxisome-related genes. Through regulating lipid turnover and oxidative balance, peroxisome pathways may contribute to maintaining cellular homeostasis and supporting the metabolic requirements of growth and immune defense. This potential role is supported by the improved antioxidant status and immune performance observed in RA-treated shrimp, including the increased T-AOC and SOD activity, the reduced MDA content, and the improved survival following *V. parahaemolyticus* challenge.

#### 4.5.5. Lysosome

The lysosomal pathway participates in autophagic processes by clearing damaged organelles and aberrant proteins to preserve cellular homeostasis, and it also facilitates intercellular crosstalk and immune modulation via lysosomal exocytosis [49]. The *npc2* gene encodes NPC intracellular cholesterol transporter 2, a vital cholesterol transporter localized in lysosomes, which can directly interact with lysosome-specific lysobisphosphatidic acid and markedly promote cholesterol trafficking across lysosomal membranes [50,51]. In the immune system, the noncanonical NF-κB signaling pathway promotes the expression of *npc2* gene, thereby regulating the transport and distribution of intracellular cholesterol, which significantly affects the immune cell function and the inflammatory responses [52]. In the present study, *npc2* gene expression showed no significant changes in the RA50 and RA150 groups, but was significantly upregulated in the RA450 group, indicating that 450 mg/kg RA may enhance cholesterol transport capacity of lysosomes by upregulating *npc2* expression. By enhancing cholesterol transport capacity of lysosome, it preserved the structural and functional integrity of the lysosomal pathway, thereby manifesting lysosomal core immune functions, such as autophagic clearance and immune communication, and then synergized with immune-related signaling pathways to sustain immune homeostasis, ultimately promoting lysosome pathway-mediated immune enhancement.

#### 4.5.6. Ether Lipid Metabolism

As vital components of cell membranes, ether lipids contribute to membrane architecture assembly and modulate T cell metabolism via signal transduction, further governing cell proliferation and inflammatory reactions [53]. The *gal3st1* gene encodes galactosylceramide sulfotransferase, which primarily catalyzes the conversion of galactosylceramide to sulfatide. As a key sulfolipid, sulfatide plays a vital role in cell membrane structure and signal transduction. The regulation of *gal3st1* expression modulates sulfolipid synthesis, thereby affecting cell membrane stability and function, as well as cellular stress responses and signal transduction [54,55]. In this experiment, *galst*, a homolog of the *gal3st1* gene, showed significantly higher expression levels in the RA150 and RA450 groups, indicating that dietary RA (150–450 mg/kg) enhanced the activity of the key sulfation step in ether lipid metabolism by upregulating *galst* expression, thereby increasing sulfatide synthesis, which stabilized the lipid environment of immune cell membranes and facilitated signal transduction, thus enhancing the immune response in shrimp.

#### 4.5.7. Glutathione Metabolism

The glutathione (GSH) metabolism pathway primarily provides antioxidant protection, detoxification, immune enhancement, and maintenance of cellular homeostasis. By binding to harmful electrophiles, GSH participates in phase II detoxification reactions, protecting aquatic animals from damage caused by environmental pollutants such as heavy metals and organic contaminants [56]. Under carbonate-alkaline stress, *Carassius auratus* enhanced the antioxidant capacity by activating glutathione metabolism to maintain cellular homeostasis while regulating ammonia excretion and energy metabolism [57]. Glutathione S-transferase 3 (GST3), a member in the glutathione S-transferase (GST) family, is a key detoxification enzyme in the glutathione metabolic pathway. It primarily protects cells from damage induced by oxidative stress and environmental pollutants by catalyzing the conjugation of glutathione with harmful electrophiles, thereby increasing aqueous solubility and facilitating excretion. In this experiment, the expression level of the *gst3* gene showed a dose-dependent trend across the RA50, RA150 and the RA450 groups with significant elevation only in the RA450 group compared to the control, indicating that RA effectively upregulated *gst3* expression and enhanced the activity of the glutathione metabolism pathway, thereby improving the resistance of *L. vannamei* to oxidative stress and pathogen invasion.

## 5. Conclusions

Dietary supplementation of 450 mg/kg RA improved the growth performance of *L. vannamei*, and supplementation of 150 and 450 mg/kg RA enhanced its immune and antioxidant capacities and increased resistance against *V. parahaemolyticus*. Improved growth, immune response and antioxidant capacity by dietary RA may be realized by regulating the pathways of arachidonic acid metabolism (*cyp2l*), glycosaminoglycan degradation (*hexb*), fatty acid biosynthesis (*hacd*), peroxisome (*ddo*, *xdh*), lysosome (*npc2*), ether lipid metabolism (*galst*) and glutathione metabolism (*gst3*). The comprehensive evaluation of RA in *L. vannamei* supports its potential as a green additive in sustainable aquaculture. The current study identified 450 mg/kg as the most effective supplementation level among the tested diets, but did not determine the definitive optimal dose; future studies should be conducted with more graded levels, as well as proteomic, metabolomic and enzymatic assays, to establish causality and evaluate long-term impacts.

## Figures and Tables

**Table 1:** Ingredients and proximate composition of experimental diets (air dry basis, g/kg).

Ingredient (g/kg)	RA0	RA50	RA150	RA450
Fish meal ^1^	180.00	180.00	180.00	180.00
Chicken meal ^1^	70.00	70.00	70.00	70.00
Soybean meal ^1^	220.00	220.00	220.00	220.00
Peanut meal	50.00	50.00	50.00	50.00
Soybean protein concentrate	50.00	50.00	50.00	50.00
Wheat flour	274.50	274.45	274.35	274.05
Corn gluten meal	50.00	50.00	50.00	50.00
Squid visceral meal	40.00	40.00	40.00	40.00
Soybean oil	15.00	15.00	15.00	15.00
Soybean lecithin	15.00	15.00	15.00	15.00
Ca(H_2_PO_4_)_2_·H_2_O	20.00	20.00	20.00	20.00
Mineral premix ^2^	5.00	5.00	5.00	5.00
Vitamin premix ^3^	5.00	5.00	5.00	5.00
Choline chloride	5.00	5.00	5.00	5.00
Vitamin C phosphate ester	0.50	0.50	0.50	0.50
Rosmarinic acid	0.00	0.05	0.15	0.45
Total	1000.00	1000.00	1000.00	1000.00
Proximate composition ^4^ (g/kg)				
Moisture	90.96	83.40	85.51	89.93
Crude protein	411.93	419.58	415.66	416.84
Crude lipid	71.88	71.95	70.29	70.82
Crude ash	78.16	74.86	80.95	85.56
Rosmarinic acid (mg/kg)	2.79	39.88	121.46	365.71

Note: ^1^ Fish meal (crude protein: 66.5%, crude lipid: 8.5%); chicken meal (crude protein: 62%, crude lipid: 11%); soybean meal (crude protein: 46%, crude lipid: 1.5%). ^2^ The mineral premix provided the following minerals per kilogram of diet: KCl, 427.5 mg; MgSO_4_, 60 mg; FeSO_4_, 125 mg; ZnSO_4_, 100 mg; C_4_H_8_CuN_2_O_4_, 25 mg; MnSO_4_, 60 mg; CoSO_4_, 1.5 mg; Na_2_SeO_3_, 0.3 mg; Ca(IO_3_)_2_, 2 mg. ^3^ The vitamin premix provided the following vitamins per kilogram of diet: vitamin A, 8000 IU; vitamin D_3_, 4000 IU; vitamin E, 100 mg; vitamin K_3_, 10 mg; vitamin B_1_, 15 mg; vitamin B_2_, 20 mg; vitamin B_6_, 40 mg; vitamin B_12_, 0.03 mg; calcium pantothenate, 40 mg; folic acid, 6 mg; nicotinamide, 60 mg; D-biotin, 0.3 mg; inositol, 200 mg. ^4^ The proximate composition and rosmarinic acid were measured values.

**Table 2:** Nucleotide sequences of the primers.

Abbreviations	Full Names	Primer Sequence (5′–3′)	Gene ID
*β-actin*	beta-actin	F:GAGCAACACGGAGTTCGTTGT	LOC113813020
		R:CATCACCAACTGGGACGACATGGA	
*hacd*	(3R)-3-hydroxyacyl-CoA dehydrogenase	F:TGATCAACAGTGCTTGACGTT	LOC113823305
		R:TGTCCTCCGGTCACTTCAAC	
*hexb*	beta-hexosaminidase subunit beta	F:TGAATACGTGGACGCCACAA	LOC113813424
		R:CATTCCCGGTTACCTGAGCA	
*cyp2l*	cytochrome P450 2L1	F:GTCCAGCCAGAAATCGGTCC	LOC113807691
		R:GGCTTGGGGAGATTGAGGAG	
*galst*	galactosylceramide sulfotransferase-like	F:GGTGCAGACCAAAGCAACTC	LOC138867334
		R:CCGTGTGAGGATTTTTGAGGC	
*npc2*	NPC intracellular cholesterol transporter 2	F:CGTCCTTAGCGAGTACCCAG	LOC113809488
		R:TCGTCAGTCAGGGACACAGA	
*ddo*	D-aspartate oxidase	F: CCCGTGTCAGAGTCGATAGC	LOC113827158
		R: ATTCCACGAAAGGACGAGCC	
*xdh*	xanthine dehydrogenase/oxidase	F: TGCCGATCTGGCCACTTATC	LOC113807596
		R: AAAGACTGCCCTTGGGTTGG	
*gst3*	glutathione S-transferase 3	F: TTATGGTGGGTAGTGCGGTG	LOC113829050
		R: ACCAGAAACCAAACCCCTATTCA	

**Table 3:** Effects of rosmarinic acid on growth performance and morphological indicators of *L. vannamei*.

Groups	RA0	RA50	RA150	RA450
IBW (g)	1.80 ± 0.07	1.80 ± 0.04	1.80 ± 0.05	1.80 ± 0.12
FBW (g)	16.60 ± 0.26 ^a^	16.74 ± 0.24 ^a^	16.86 ± 0.17 ^a^	17.41 ± 0.25 ^b^
SR (%)	96.25 ± 2.50	93.75 ± 2.50	95.63 ± 3.15	95.63 ± 3.15
FI (g)	21.74 ± 0.22	21.84 ± 0.14	21.57 ± 0.11	21.67 ± 0.38
WG (%)	822.24 ± 14.47 ^a^	829.89 ± 13.45 ^a^	836.66 ± 9.20 ^a^	867.06 ± 13.78 ^b^
FCR	1.46 ± 0.03 ^b^	1.46 ± 0.02 ^b^	1.43 ± 0.02 ^b^	1.39 ± 0.01 ^a^
HSI (%)	3.91 ± 0.31	3.86 ± 0.37	4.11 ± 0.28	4.12 ± 0.31
CF (g/cm^3^)	1.08 ± 0.06	1.10 ± 0.08	1.09 ± 0.05	1.10 ± 0.06
MY (%)	48.94 ± 5.23	49.06 ± 3.70	49.49 ± 2.57	49.98 ± 3.56

Note: SR: survival rate, WG: weight gain, FI: feed intake, FCR: feed conversion ratio, CF: condition factor, HSI: hepatosomatic index, MY: meat yield. Different letters within the same row indicate significant differences (*p* < 0.05).

**Table 4:** Effects of rosmarinic acid on whole body composition of *L. vannamei* (fresh weight, g/kg).

Groups	RA0	RA50	RA150	RA450
Moisture	747.81 ± 20.70	736.01 ± 10.16	742.65 ± 7.64	748.18 ± 9.87
Crude protein	190.25 ± 5.24	195.76 ± 2.16	194.26 ± 1.95	192.37 ± 3.92
Crude lipid	14.12 ± 1.07	14.94 ± 1.41	14.38 ± 1.33	14.25 ± 1.19
Crude ash	26.80 ± 2.09	26.27 ± 1.39	26.21 ± 3.22	26.25 ± 4.75

**Table 5:** Effects of rosmarinic acid on biochemical parameters of serum and hepatopancreas in *L. vannamei*.

Groups	RA0	RA50	RA150	RA450
Serum				
TP (gprot/L)	52.77 ± 5.69	56.64 ± 5.47	57.14 ± 5.45	58.13 ± 5.62
GLU (mmol/L)	1.12 ± 0.13	1.21 ± 0.10	1.23 ± 0.16	1.16 ± 0.15
T-CHO (mmol/L)	1.51 ± 0.19	1.66 ± 0.13	1.74 ± 0.18	1.70 ± 0.11
TG (mmol/L)	0.51 ± 0.06	0.47 ± 0.06	0.51 ± 0.07	0.54 ± 0.06
ACP (King unit/100 mL)	4.19 ± 0.59 ^a^	6.12 ± 0.46 ^b^	6.45 ± 0.66 ^b^	6.22 ± 0.56 ^b^
AKP (King unit/100 mL)	1.88 ± 0.18 ^a^	2.12 ± 0.18 ^a^	4.34 ± 0.51 ^c^	2.64 ± 0.27 ^b^
LZM (U/mL)	72.07 ± 3.12 ^a^	77.48 ± 6.24 ^ab^	88.29 ± 6.24 ^b^	100.90 ± 8.26 ^c^
ACH50(U/mL)	23.02 ± 1.45 ^a^	33.72 ± 2.61 ^b^	31.71 ± 1.51 ^b^	32.20 ± 2.29 ^b^
Hepatopancreas				
T-AOC (mmol/gprot)	0.48 ± 0.05 ^a^	0.51 ± 0.05 ^a^	0.62 ± 0.08 ^b^	0.66 ± 0.07 ^b^
MDA (nmol/mgprot)	1.58 ± 0.22 ^b^	1.60 ± 0.16 ^b^	1.27 ± 0.14 ^a^	1.25 ± 0.21 ^a^
SOD (U/mgprot)	9.45 ± 1.01 ^a^	10.43 ± 0.59 ^ab^	10.46 ± 0.65 ^ab^	11.22 ± 0.84 ^b^

Note: Different letters within the same row indicate significant differences (*p* < 0.05).

**Table 6:** Effects of rosmarinic acid on the intestinal morphology of *L. vannamei*.

Groups	RA0	RA50	RA150	RA450
Villus height (μm)	45.00 ± 3.92 ^a^	50.50 ± 4.81 ^ab^	47.97 ± 4.15 ^ab^	51.11 ± 4.91 ^b^
Villus width (μm)	37.45 ± 3.66 ^a^	41.79 ± 4.58 ^ab^	47.23 ± 4.73 ^c^	45.82 ± 3.84 ^bc^
Muscular thickness (μm)	18.18 ± 1.85 ^a^	19.62 ± 1.71 ^ab^	22.48 ± 2.11 ^c^	21.27 ± 2.02 ^bc^

Note: Different letters within the same row indicate significant differences (*p* < 0.05).

**Table 7:** Effects of rosmarinic acid on B-cell, R-cell, and F-cell number in hepatopancreas of *L. vannamei*.

Groups	RA0	RA50	RA150	RA450
B cell (number/tubule)	5.83 ± 1.19	5.93 ± 1.49	6.36 ± 1.50	6.60 ± 1.58
R cell (number/tubule)	23.90 ± 3.96 ^a^	26.50 ± 3.57 ^a^	36.00 ± 4.42 ^b^	45.00 ± 6.11 ^c^
F cell (number/tubule)	13.21 ± 1.81 ^ab^	13.00 ± 3.07 ^a^	15.38 ± 3.43 ^bc^	16.80 ± 2.39 ^c^

Note: Different letters within the same row indicate significant differences (*p* < 0.05).

## Data Availability

Data are contained within this article.
